# Ascites Increases Expression/Function of Multidrug Resistance Proteins in Ovarian Cancer Cells

**DOI:** 10.1371/journal.pone.0131579

**Published:** 2015-07-06

**Authors:** Lihong Mo, Vendula Pospichalova, Zhiqing Huang, Susan K. Murphy, Sturgis Payne, Fang Wang, Margaret Kennedy, George J. Cianciolo, Vitezslav Bryja, Salvatore V. Pizzo, Robin E. Bachelder

**Affiliations:** 1 Department of Pathology, Duke University Medical Center, Durham, North Carolina, 27710, United States of America; 2 Faculty of Science, Masaryk University, Kotlarska 2, 61137, Brno, Czech Republic; 3 Department of Obstetrics and Gynecology, Duke University Medical Center, Durham, North Carolina, 27710, United States of America; Columbia University, UNITED STATES

## Abstract

Chemotherapy resistance is the major reason for the failure of ovarian cancer treatment. One mechanism behind chemo-resistance involves the upregulation of multidrug resistance (MDR) genes (ABC transporters) that effectively transport (efflux) drugs out of the tumor cells. As a common symptom in stage III/IV ovarian cancer patients, ascites is associated with cancer progression. However, whether ascites drives multidrug resistance in ovarian cancer cells awaits elucidation. Here, we demonstrate that when cultured with ascites derived from ovarian cancer-bearing mice, a murine ovarian cancer cell line became less sensitive to paclitaxel, a first line chemotherapeutic agent for ovarian cancer patients. Moreover, incubation of murine ovarian cancer cells *in vitro* with ascites drives efflux function in these cells. Functional studies show ascites-driven efflux is suppressible by specific inhibitors of either of two ABC transporters [Multidrug Related Protein (MRP1); Breast Cancer Related Protein (BCRP)]. To demonstrate relevance of our findings to ovarian cancer patients, we studied relative efflux in human ovarian cancer cells obtained from either patient ascites or from primary tumor. Immortalized cell lines developed from human ascites show increased susceptibility to efflux inhibitors (MRP1, BCRP) compared to a cell line derived from a primary ovarian cancer, suggesting an association between ascites and efflux function in human ovarian cancer. Efflux in ascites-derived human ovarian cancer cells is associated with increased expression of ABC transporters compared to that in primary tumor-derived human ovarian cancer cells. Collectively, our findings identify a novel activity for ascites in promoting ovarian cancer multidrug resistance.

## Introduction

Operative tumor debulking is performed mainly on stage I/II ovarian cancer patients. This surgical procedure for advanced stage disease (III to IV) is not always possible, especially in women whose disease is extensive [1]. Therefore, chemotherapy is the primary tool for blocking dissemination of cancer cells when clinicians treat patients at advanced cancer stages. Compared to normal cells, actively proliferating cancer cells are more susceptible to a variety of cytotoxic drugs targeting different cellular processes, including DNA alkylating agents, antimetabolites, intercalating agents and mitotic inhibitors [2].

The first-line chemotherapy for ovarian cancer has remained unchanged over the last decade, with the therapeutic backbone consisting of a platinum agent (generally carboplatin) and a taxane (generally paclitaxel) [3]. Second-line chemotherapies are considered when the patients are unresponsive to first-line drugs. A number of antineoplastic agents have demonstrated sufficient biological activity to be considered rational second-line choices, such as doxorubicin, etoposide, gemcitabine, ifosfamide, or cyclophosphamide [4].

Chemo-resistance, characterized by a reduced ability of chemotherapy to inhibit tumor growth over time, is the single most common reason for discontinuing chemotherapy treatment. Ovarian cancer recurrence is a direct outcome of chemo-resistance, occurring in more than 80% of high-grade serous ovarian cancer patients [3, 5]. The mechanisms behind chemo-resistance include: 1) upregulation of multidrug resistance (MDR) genes that effectively transport drugs out of the cell; 2) alteration of drug-metabolizing enzymes, such as those in the glutathione s-transferase family (GST); 3) escape from apoptosis and increased DNA repair due to mutated tumor suppressor genes [p53, breast cancer 1/2 (BRCA1/2), and ataxia telangiectasia mutated (ATM) genes] [2]; and 4) impairment of mitotic spindle checkpoint leading to resistance to microtubule inhibitors [6].

A large family of 50 different ATP-binding cassette (ABC) proteins (ABC transporters) have been documented to efflux cytotoxic molecules, reducing the intracellular drug concentration [7, 8]. Among the ABC transporters associated with chemo-resistance of ovarian cancer, the *MDR1* gene, which encodes P-glycoprotein (P-gp; MDR1, ABCB1), is the most frequently studied mechanism. Other common ABC transporters include: the MDR-associated protein 1 (MRP1, ABCC1) and the breast cancer resistance protein (BCRP, ABCG2) [2]. Short term incubation of ovarian cancer cells with chemotherapeutic regimens (e.g. doxorubicin, cisplatin and paclitaxel) at their clinical concentrations [9] increases MDR1 expression levels. Notably, recurrent ovarian cancers demonstrate significantly increased MDR1 compared to primary ovarian cancers, with the recurrent patients receiving platinum-taxane therapy as a standard of care after the diagnosis of their primary cancer [10]. Similar to MDR1, MRP1 is detected in untreated primary ovarian tumors at varying levels [11] and found upregulated after a stepwise induction of cisplatin resistance in ovarian cancer cell lines *in vitro* [12]. BCRP is inducible in ovarian cancer cell lines by long-term incubation with topotecan and confers resistance to topotecan and mitoxanthrone [13, 14].

Ascites is a common symptom in stage III/IV ovarian cancer patients and correlates with a poor prognosis [15]. Malignant ascites is known to protect human ovarian cancer cells from TRAIL-induced apoptosis leading to a shorter disease-free survival of patients [16, 17]. However, little is known about the relationship between the presence of ascites and chemo-resistance in ovarian cancer. In this study, we investigate how ascites affects ovarian cancer cells in their responses to paclitaxel and docetaxel, leading taxane drugs employed by clinicians in ovarian cancer treatment [3].

## Materials and Methods

### Cell line and reagents

ID8, a mouse epithelial ovarian cancer cell line [18], was a kind gift from Dr. Kathy Roby at Kansas University Medical Center. Mycoplasma contamination screening using Gen-Probe nucleic acid hybridization was performed by the Duke Cancer Institute Cell Culture Facility in April 2010. ID8 cells were maintained in DMEM (high glucose, Gibco-Life Technologies [Gibco]; Carlsbad, CA) containing 4% fetal bovine serum, penicillin (100 units/mL) + streptomycin (100 μg/ml) (Invitrogen-Life Technologies [Invitrogen]; Carlsbad, CA). HA1 and HA2 were developed by SV40 T antigen-immortalization of human ovarian cancer cells obtained from ascites [HA1 line derived from ascites (6116) from primary surgery serous carcinoma, poorly differentiated; HA2 line derived from ascites (OV 186) from primary surgery serous papillary cystadenocarinoma]. TD cells were developed by SV40 immortalization of human ovarian cancer cells from primary surgery high grade serous adenocarcinoma tissue (primary peritoneal carcinoma) (6114)]. These cell lines were maintained in DMEM (high glucose, Gibco) containing 10% fetal bovine serum and 1X penicillin streptomycin. De-identified tumor and ascites samples used to create the immortalized cell lines were collected, following donor's provision of written informed consent, by the Duke Gynecologic Oncology Tumor Bank (Pro00013710) and used for this study under Duke IRB protocol Pro00027325. Paclitaxel (T7191) and docetaxel (01885) were purchased from Sigma-Aldrich, St. Louis, MO. Human ovarian cancer cell lines were maintained for a maximum of two passages prior to analysis.

### Murine ascites preparation

This study was carried out in strict accordance with the recommendations in the Guide for the Care and Use of Laboratory Animals of the National Institutes of Health. The protocol was approved by the Institutional Animal Care & Use Committee at Duke University (protocol number: A295-12-11). All efforts were made to minimize suffering. Female 6–8 week-old C57BL/6 mice (Charles River; Raleigh, NC) were used. ID8 cells (1-10x10^6^) were injected into the mouse peritoneum. After ascites accumulation, mice were euthanized. Peritoneal fluid was collected and centrifuged twice at 500 x g for 5 min to separate the cellular and acellular fractions. Acellular fractions from multiple mice were pooled and filtered through 0.22 μm sterile filters. The 50% ascites treatment conditions were normalized to normal culture conditions with regard to FBS, glucose and antibiotic concentrations.

### Flow cytometric analysis with Green Efflux ID Dye

The functions of the multidrug resistance proteins MDR1, MRP and BCRP were analyzed by eFluxx-ID Green Multidrug Resistance Assay Kit (ENZ-51029-K100, Enzo Lifesciences, Farmingdale, NY) according to the manufacturer’s instructions. Briefly, single cell suspensions harvested by trypsin were counted, equal numbers of cells (500,000/condition) were resuspended in full media containing specificinhibitors or their combinations (or DMSO as diluent control) andincubated for 10 min at 37°C. Sources/final concentrations of inhibitors are as follows: Verapamil (MDR1 inhibitor; Sigma V4629; 40 μM); MK-571 (MRP1 inhibitor; Sigma M7571; 100 μM); Novobiocin (BCRP inhibitor; Sigma N1628; 200 μm). Green dye was then added and incubated at 37°C for 40 min. Cells were washed once in PBS before FACS data acquisition using a Guava Easycyte Plus instrument (Millipore, Billerica, MA), 7-AAD was used to exclude dead cells from the analysis. Data were analyzed using FlowJo_V10 software (Ashland, OR).

### Calculation of Multidrug resistance activity factor

The multidrug resistance activity factor (MAF) for each transporter, was calculated using the following formulas:

$${\text{MAF}}_{\text{MDR}1}=100\;\text{x}\;\left({\text{F}}_{\text{MDR}1}\text{-}\;{\text{F}}_{0}\right)/{\text{F}}_{\text{MDR}1}$$

$${\text{MAF}}_{\text{MRP}}=100\;\text{x}\;\left({\text{F}}_{\text{MRP}}\text{-}\;{\text{F}}_{0}\right)/{\text{F}}_{\text{MRP}}$$

$${\text{MAF}}_{\text{BCRP}}=100\;\text{x}\;\left({\text{F}}_{\text{BCRP}}\text{-}\;{\text{F}}_{0}\right)/{\text{F}}_{\text{BCRP}}$$

where F is the geometric mean of fluorescence intensity (GMFI), F_0_ – without inhibitor, F_MDR1_ – with MDR1 inhibitor, F_MRP_ – with MRP inhibitor, F_BCRP_ – with BCRP inhibitor

### Rhodamine 123 retention assay

Drug efflux function was determined in untreated and ascites-treated ID8 cells by rhodamine 123 retention assays [18]. ID8 cells obtained from normal culture, from 7 day ascites pretreatment culture or from ascites *in vivo* were seeded at 40,000 cells per well in 6-well plates and allowed to attach overnight. These cells were then incubated with Rhodamine 123 for 30 min. Cells from each condition were then washed with PBS and incubated with regular medium for 2.5 h. Fluorescent pictures were taken at an excitation λ = 485 nm and emission λ = 530 nm using BioTek Cytation3 Imager (Winooski, VT) at 0 min (after dye removal and PBS wash) and at 2.5h hours after Rhodamine 123 removal. Experiments were independently repeated three times.

### Real-Time quantitative RT-PCR

Total RNA from cells was extracted using Qiagen RNeasy Micro kit according to manufacturer’s protocol (Qiagen; Valencia, CA) and treated with RNase-free DNase to remove any residual genomic DNA. Single-stranded cDNAs were synthesized by incubating total RNA (1 μg) with RNase H-reverse transcriptase (500 U), oligo-(dT)12–18 primer (100 nM), dNTPs (1 mM), and RNase-inhibitor (40 U) at 42°C for 1 h in a final volume of 20 μl. Transcript First Strand cDNA Synthesis Kit was purchased from Roche Applied Science (Indianapolis, IN). Q-PCR primers for mouse *Abcc1*, *Abcb1a*, *Abcb1b* and *beta-Actin* were purchased from Origene (Rockville, MD). Quantitative real-time PCR was performed to compare expression levels of each total RNA sample. Real-time PCR on the Mx3005P QPCR System (Stratagene, La Jolla, CA) was performed in the presence of 12.5 μl VeriQuest Fast SYBR Green qPCR Master mix(2x) (USB, Cleveland, OH) and 2 μl cDNA, and H_2_O was added to a final volume of 25μl. Real-time PCR was performed with an initial denaturation step of 5 min at 95°C, followed by 40 cycles of 3 s at 95°C, 30 s at an annealing temperature at 60°C. PCR products were monitored in real time by measuring the increase in fluorescence caused by the binding of SYBR Green I Dye. Significance was analyzed using the software package MxPro QPCR Software (Stratagene, La Jolla, CA).

### Western Blots

Cells were harvested using trypsin-EDTA and washed with PBS. Harvested cells were incubated in total lysis buffer (50mM Tris pH 7.5, 1% SDS) plus protease and phosphatase inhibitor cocktail (Halt, Thermo Scientific). Protein concentrations were determined by BCA assay. Equivalent amounts of protein were subjected to SDS-polyacrylamide gel electrophoresis (PAGE) and immunoblotted. Blots were incubated with the following primary antibodies, followed by the appropriate species IRDye-conjugated secondary antibody (Life Technologies, Carlsbad, CA): MDR1 (BIOSS; Cat# 1468R); MRP1 (Biorbyt Cat# ORB76148); BCRP (Biorbyt Cat#ORB10178); beta actin (Sigma Cat# SAB5500001). MDR1, MRP1, BCRP proteins were detected using Odyssey infrared imaging system (LI-COR, Lincoln, NE). Protein bands were quantified using Image J software (NIH, Bethesda, MD), and the relative ratio of the protein of interest to beta actin loading control was calculated.

### Proliferation assays

Cells were seeded at 2.5x10^3^ cells per well in 96-well plates and allowed to grow overnight. Cells were washed once with serum-free DMEM. The cells were then incubated with different concentrations of paclitaxel or docetaxel diluted in 200 μl DMEM for 24 h. The ascites treated ID8 cells or ID8 cells harvested from ascites received the treatments diluted in 200 μl 50% ascites supernatant. At 18 h, 0.5 μCi [^3^H]-thymidine (Perkin-Elmer, Waltham, MA) was added to each well. After 8 h, the media was removed and the cells were dissociated in 10X trypsin-EDTA and harvested using a 12-channel microharvester (Skatron Instruments, Norway). The samples were counted in a liquid scintillation spectrophotometer. All conditions were in quadruplicate, and each experiment repeated at least twice.

### 7-AAD staining

ID8 cells from different pre-treatments were seeded at 4x10^4^ cells per well and allowed to grow overnight in 6-well plates. They were washed once with serum-free DMEM. The cells were then incubated with different concentrations of paclitaxel or docetaxel diluted in 2 ml DMEM for 4 days. Ascites treated ID8 cells or ID8 cells harvested from ascites received the treatments diluted in 2ml 50% ascites supernatant. At the end of treatments, cells were harvested by trypsinization and stained with 7-AAD (BD Biosciences) according to the manufacturer’s protocol followed by analysis on a Guava Easycyte Plus instrument (Millipore) and analyzed by FlowJo_V10 software.

### Statistical analysis

Graph visualization, data transformation and statistical analysis (two-tailed unpaired t-test or Two way – ANOVA) were performed using Prism (version 5.0, GraphPad Software) and Microsoft Office Excel (version 2010, Microsoft).

## Results

### Ascites increases ID8 cell chemotherapy resistance

**Fig 1:**
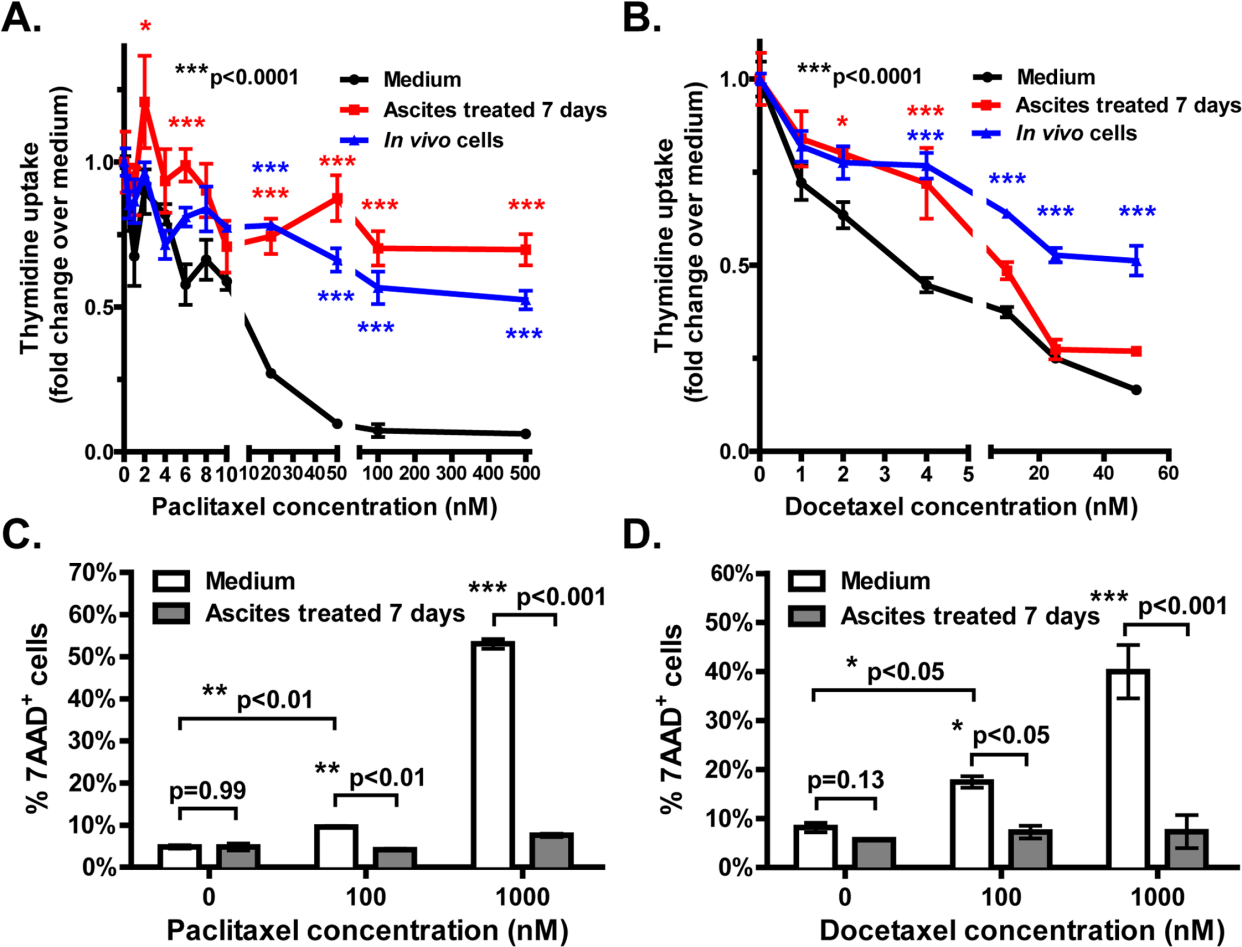
Ascites derived from ovarian cancer-bearing mice increases chemo-resistance of ID8 cells. Murine ovarian cancer cells (ID8) from three different sources were studied: 1) ID8 cells from normal culture medium (Medium), 2) ID8 cells freshly isolated from ascites fluid in a syngeneic model (*In vivo* cells), and 3) ID8 cells treated for 7 days *in vitro* with ascites supernatant (Ascites treated 7 days). Each of these cell populations was exposed to paclitaxel**(A)** or docetaxel**(B)** at the indicated concentration for 24 h and [^3^H]-thymidine incorporation was determined. Three independent experiments were performed and a representative result is shown. Error bar represents SD of quadruplicates in each condition. * indicates p<0.05, *** p<0.001, Two way ANOVA. **A**. ID8 cells pre-treated with acellular ascites for 7 days (Ascites treated 7 days) or isolated from ascites (*in vivo* cells) have increased resistance to Paclitaxel compared to ID8 cells from normal medium. **B**. ID8 cells pre-treated with acellular ascites for 7 days (Ascites treated 7 days) have increased resistance to docetaxel at 2 and 4 nM compared to ID8 cells from normal culture. ID8 cells isolated from ascites (*in vivo* cells) have increased resistance to docetaxel compared to ID8 cells from normal medium (Medium). **C-D**. 7-AAD uptake was measured by flow cytometric analysis. The percent 7-AAD positive cells from three independent experiments is shown for paclitaxel (**C**) and docetaxel (**D**)-treated cells. Error bars represent SD. * indicates p<0.05, ** p<0.01, *** p<0.001, Two way ANOVA. ID8 cells pre-treated with acellular ascites for 7 days (Ascites treated 7 days) are more resistant to chemotherapy-induced cell death (*i*.*e*.,exhibit fewer 7-AAD+ cells) than ID8 cells from normal culture.

We tested the effect of ascites on ovarian cancer cell resistance to paclitaxel, a first line chemotherapeutic agent. Comparison was performed between 1) ID8 cells grown in normal culture medium, 2) ID8 cells freshly isolated from ascites fluid in a syngeneic model, and 3) ID8 cells treated for 7 days *in vitro* with ascites supernatant derived from ID8-bearing mice. Chemotherapy dosing was optimized for a range to achieve 0 to 95% cell growth inhibition, as measured by [^3^H]-thymidine incorporation. Both the ID8 cells treated for 7 days with ascites supernatant and ID8 cells freshly isolated from ascites fluid were more resistant to paclitaxel than ID8 cells cultured in normal medium, as measured by [^3^H]-thymidine incorporation assay (Fig 1A). To validate the hypothesis that ascites drives ovarian cancer cell chemo-resistance, we next studied the response of ovarian cancer cells (+/- ascites treatment) to another chemotherapeutic reagent in the taxane family (docetaxel). Notably, ID8 cells treated for 7 days with ascites supernatant, as well as ID8 cells freshly isolated from ascites fluid were significantly more resistant to docetaxel than ID8 cells from normal culture (Fig 1B).

The above data indicate that ascites increases ovarian cancer cell chemo-resistance, as assessed using a [^3^H]-thymidine incorporation assay, which is a measure of cell proliferation. We next sought to determine whether ascites protects ovarian cancer cells from chemotherapy-induced cell death by performing 7-AAD staining. As shown in Fig 1C and 1D, ID8 cells treated for 7 days with cell-free ascites were protected from both paclitaxel- and docetaxel- induced cell death, providing further evidence that ascites supernatant promotes ovarian cancer chemo-resistance.

### Ascites drives efflux in ID8 cells

**Fig 2:**
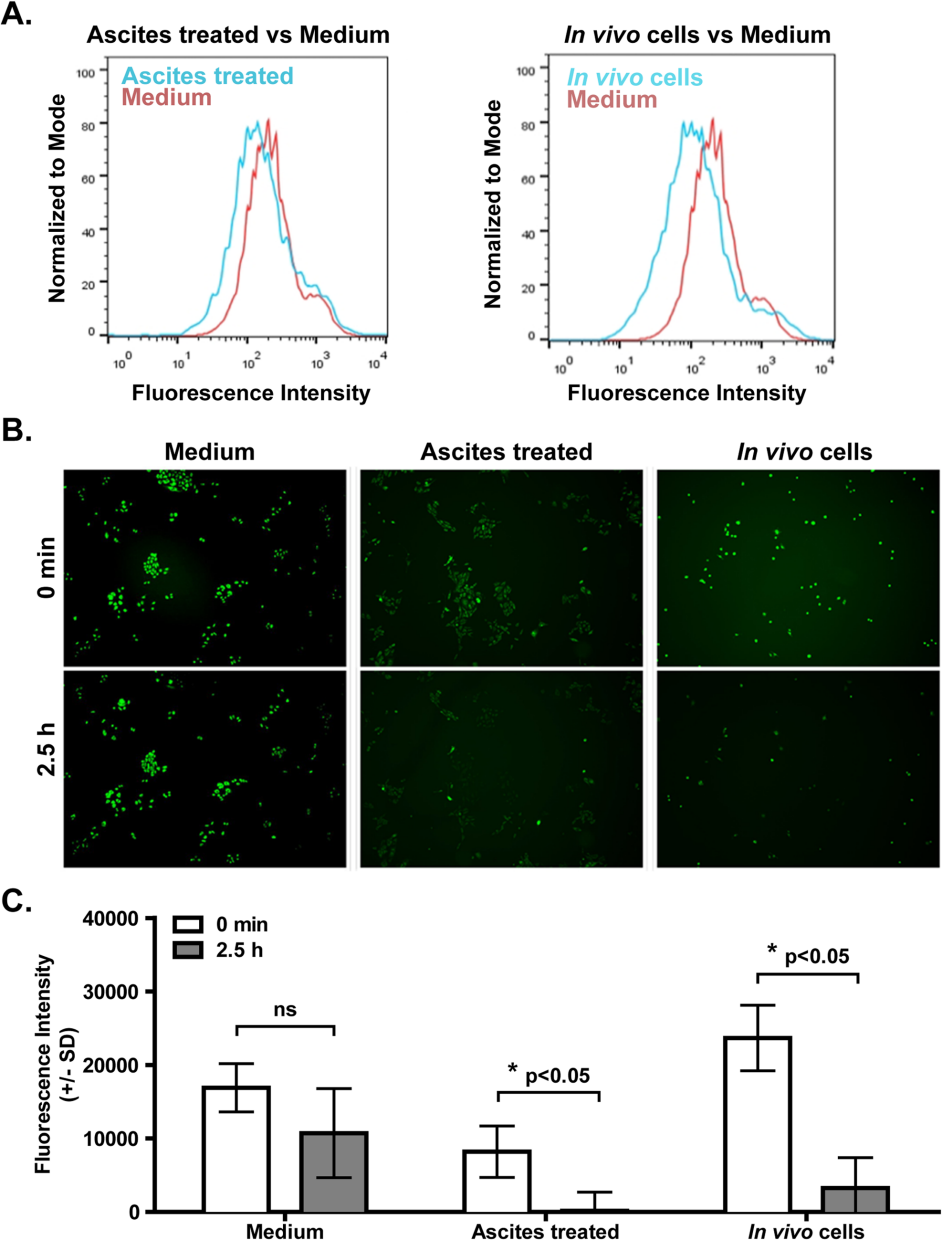
Ascites increases efflux function in ID8 cells. (**A**). Efflux function was measured by eFFlux ID Green dye assay. ID8 cells isolated from normal culture, obtained after 7 day ascites treatment, or isolated from ascites (*in vivo* cells) were incubated with eFFlux ID Green dye for 40 min, allowing the cells to uptake and efflux this dye. ID8 cells pre-treated with ascites retained less dye compared to ID8 cells from normal culture, indicating an increased efflux function. (**B**). Efflux function was measured by Rhodamine 123 assay. ID8 cells obtained from normal culture, from 7 day ascites pretreatment culture or from ascites *in vivo* were incubated with Rhodamine 123 for 30 min. Then cells from each condition were washed with PBS and incubated with regular medium for 2.5 h. Fluorescent pictures were taken at 0 min (after dye removal and PBS wash) and at 2.5h hours after Rhodamine 123 removal. Quantification of the fluorescence intensity (minus background) in each group is shown in (**C**). Three independent experiments were performed and a representative result is shown. Error bar represents SD of fluorescent intensity measuring each cell in each image. * indicates p<0.05, Student’s t-test.

We next investigated efflux function in ascites-treated ovarian cancer cells using two drug efflux assays (eFFlux ID green dye and rhodamine 123) [18]. The eFFlux ID green assay measures fluorescence intensity after incubating cells with fluorescent dye. Fig 2A shows reduced fluorescence intensity in both ascites pre-treated ID8 cells and ID8 cells isolated from ascites (syngeneic mouse model) compared to fluorescence intensity in untreated cells. These results suggest that ascites promotes efflux function in ovarian cancer cells. We next measured retention of rhodamine 123 dye in ID8 cells (+/- ascites treatment). As shown in Fig 2B and 2C, 2.5h after rhodamine removal, untreated ID8 cells had retained dye. In contrast, significantly reduced levels of dye were detected in ascites-preincubated ID8 cells, supporting the conclusion that ascites-treated tumor cells exhibit increased efflux. Together, these results suggest that ascites drives ovarian cancer efflux mechanisms.

### Ascites treatment increases expression of multidrug resistance-related transporters

**Fig 3:**
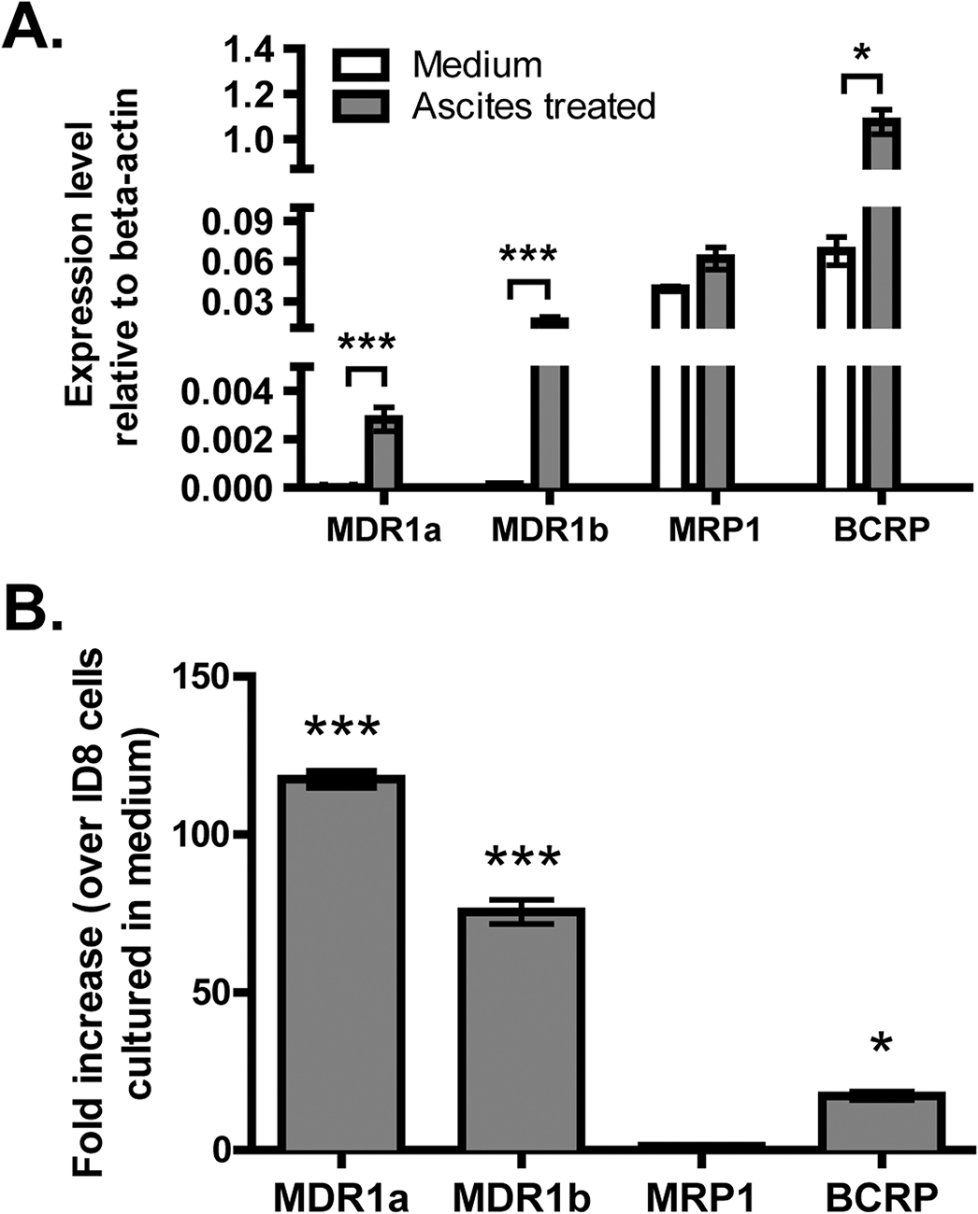
Ascites treatment increases expression of MDR1a/b and BCRP in ID8 cells. Total RNA was harvested from ID8 cells in normal culture as well as from ID8 cells pre-treated with ascites for 7 days. Expression levels of the indicated genes (relative to beta actin) were determined by real-time PCR(**A**). Fold increases in gene expression (ascites treated ID8 cells over normal ID8 cells) are shown in **B**. Three independent experiments were performed and error bar represents SD. * indicates p<0.05 and *** p<0.001, Student’s t-test.

We studied the expression of three ABC transporter genes (MDR1, MRP1 and BCRP) that are commonly involved in cancer chemo-resistance. We performed quantitative real-time PCR (q-PCR) on RNA harvested from ID8 cells and ID8 cells pre-treated with ascites. Untreated ID8 cells expressed both MRP1 and BCRP mRNA (Fig 3A). After treatment with ascites *in vitro* for 7 days, ID8 cells exhibited significantly increased expression of MDR1a (118- fold), MDR1b (75-fold), and BCRP (17-fold)(Fig 3A and 3B).

### Inhibitors of multidrug resistance-related transporters reduce efflux efficiency in ascites-associated ovarian cancer cells

**Fig 4:**
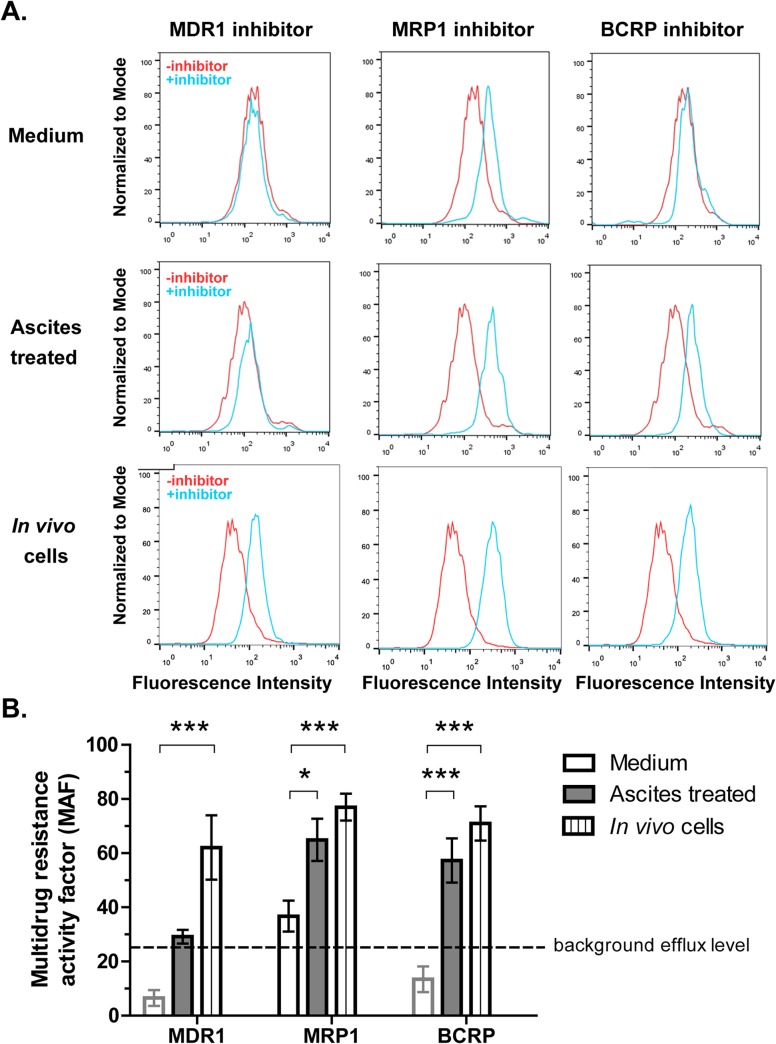
BCRP and MRP1 inhibitors suppress efflux function in ascites treated ID8 cells. (**A**). ID8 cells from normal culture, ascites pre-treatment culture, or ascites (*in vivo* cells) were treated with or without inhibitors targeting MDR1, MRP1 or BCRP for 10 min and then incubated with eFFlux ID Green dye for 30 min. fluorescence intensity of each condition was measured by flow cytometry. Multidrug resistance activity factor (MAF) was calculated for each sample, and mean MAF (+/- SD from triplicate samples) is shown for each condition in B. MAF values falling below background are indicated by gray border. Statistically significant increases in MAF between ascites-treated and *in vivo* ascites cells (compared to untreated cells) are indicated. Error bars represent SDs from three independent experiments. * indicates p<0.05, Student’s t-test.

After demonstrating upregulated MDR-related ABC transporters in ascites treated ovarian cancer cells, we next sought to determine whether specific inhibitors of these transporters prevent efflux in these cells. We studied three MDR transporter inhibitors: verapamil (MDR1 inhibitor), MK-571 (MRP1/2 inhibitor), and Novobiocin (BCRP inhibitor). First, inhibitor effects on efflux function in ID8 cells cultured in normal medium was assessed. Inhibitors were added to cells for 10 minutes before the addition of eFFlux ID green dye. As shown in Fig 4A and 4B, only the MRP1 inhibitor increased fluorescence intensity of efflux ID green- labeled ID8 cells. These results indicate that untreated ID8 ovarian cancer cells support MRP1-dependent efflux, but not MDR1 or BCRP-dependent efflux. Next we studied effects of these inhibitors on efflux function in both ascites treated ID8 cells and in ID8 cells isolated directly from ascites (*in vivo* cells). MDR1 inhibitor did not increase fluorescence in ID8 cells pre-incubated with ascites for 7 days *in vitro*. However, MDR1 inhibitor did increase fluorescence in ID8 cells isolated from ascites *in vivo* (Fig 4A and 4B). Addition of either MRP1 inhibitor or BCRP inhibitor significantly increased fluorescence (MAF) in both ascites-treated ID8 cells and in ascites cells (*in vivo*) (Fig 4B). We also showed that combinations of these inhibitors did not increase fluorescence intensity of efflux ID green-labeled ID8 cells or ascites-treated cells over the fluorescence intensity obtained with single inhibitors, suggesting that activity of these individual transporters was not compensated for by other transporters (data not shown). Collectively, these data suggest that ascites promotes MRP1 and BCRP-dependent efflux in ID8 ovarian cancer cells.

### Human ovarian cancer cells obtained from ascites, but not tumor-derived ovarian cancer cells, exhibit efflux function

**Fig 5:**
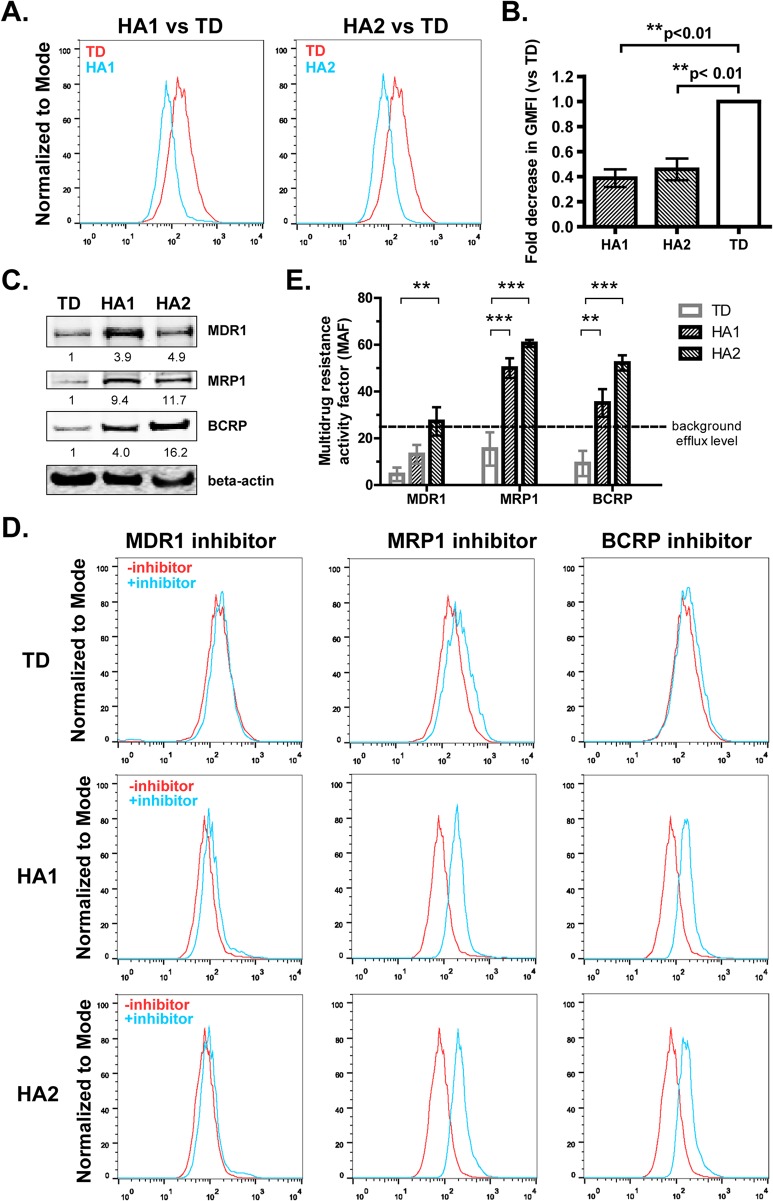
Human ovarian cancer cells derived from ascites exhibit increased efflux compared to cells derived from the primary tumor. **A and B.** Immortalized human ovarian cancer cells derived from patient ascites (HA1 and HA2 cells) or primary tumor site (TD cells) were incubated with eFFLux ID green dye for 40 min. Fluorescence intensity for each condition was measured by flow cytometry. Histogram of each cancer cell line derived from patient ascites (HA1 or HA2; blue) is overlaid with histogram of the primary tumor-derived line (TD; red)). **B.** Mean fluorescence intensity (MFI) was calculated for each line from three independent experiments. Fold decrease in geometric mean fluorescence intensity (GMFI) (relative to tumor-derived line) was calculated for each cell line in three independent experiments. Results are reported as the mean fold decrease from three independent trials treated (+/- SD). **C.** Total cellular extracts were obtained from a primary tumor-derived human ovarian cancer cell line (TD) and from each of two ascites-derived human ovarian cancer cell lines (HA1, HA2). Equivalent amounts of extracted proteins were subjected to SDS-PAGE and immunblotted with antibodies specific for MDR1, MRP1, BCRP, or beta actin, followed by the appropriate species IRdye-conjugated secondary antibody. Protein bands were detected by Odyssey infrared imaging. Protein bands were quantified using Image J software (NIH). Ratios of the indicated protein to beta actin are shown. **D and E.** Ascites-derived and tumor-derived human cell lines were incubated with or without inhibitors targeting MDR1, MRP or BCRP for 10 min and then incubated with eFFlux ID Green dye for 30 min. Samples were analyzed by flow cytometry. Histograms (+ inhibitor vs – inhibitor) were overlaid for each line (**D**). **E.** Multidrug resistance activity factor (MAF) was calculated for each sample, and mean MAF (+/- SD) from three independent experiments is shown for each condition in E. MAF values falling below background are indicated in gray. Statistically significant increases in MAF between ascites-treated and *in vivo* ascites cells (compared to untreated cells) are indicated. * indicates p<0.05. ** indicates p<.01, Student’s t-test.

Next, we sought to extend our findings to human ovarian cancer cells. We studied immortalized human ovarian cancer cells derived from patient ascites (HA1, HA2 cells) or from the primary tumor site (TD cells). Using the eFFlux ID green assay, we showed that HA1 and HA2 cells exhibited higher efflux than TD cells (Fig 5A and 5B), lending support to our finding that ascites drives the efflux function of ovarian cancer cells. Western blotting was performed to investigate efflux protein expression in these tumor-derived and ascites-derived human ovarian cancer cells. As shown in Fig 5C, expression of MDR1, MRP1 and BCRP was significantly higher in ascites-derived tumor cell lines (HA1, HA2) than in a primary tumor-derived cell line (TD). To determine which efflux proteins were functional in these cells, we next investigated effects of MDR inhibitors on efflux in tumor-derived and ascites-derived human ovarian cancer cells. MRP1 and BCRP inhibitors, but not an MDR1 inhibitor, suppressed dye efflux in HA1 and HA2 cells significantly. In contrast, inhibitors of MDR1, MRP1 and BCRP did not impact dye retention in TD cells (Fig 5D and 5E). We showed that combinations of these inhibitors did not increase fluorescence intensity of TD cells (data not shown), suggesting that activity of individual transporters was not compensated for by other transporters. These findings indicate ascites-derived human ovarian cancer cells, but not primary tumor-derived cells, support ABC transporter-dependent efflux.

## Discussion

Ovarian cancer cells acquire resistance to chemotherapy after short-term [7] or long-term [12, 14] treatment with chemotherapeutic drugs. Both malignant ascites [15] and chemo-resistance[19] have been associated with reduced survival of ovarian cancer patients. In this study, we demonstrate that murine ovarian cancer cells (ID8) exhibit increased taxane chemo-resistance when exposed to ascites *in vitro* or *in vivo* (compared to ID8 cells cultured in normal medium). To our knowledge, our work is the first to directly demonstrate inducible chemo-resistance by ascites supernatant.

To date, no study has investigated an association between the presence of ascites [15] and MDR transport [20–22], although each is an independent risk factor in predicting poor prognosis for ovarian cancer patients. Studies have characterized the expression level of the MDR transporter on primary or recurrent ovarian cancer tissues [10] or MDR1 polymorphism and ovarian cancer progression or survival [23]. Our study is novel in suggesting that ascites is capable of driving the expression and function of MDR transporters. Specifically, we show that ascites pre-treated ID8 cells, but not untreated cells, are capable of effluxing two different dyes (Fig 2). Ascites-associated efflux in murine ovarian cancer cells was inhibited by specific inhibitors of either of two ABC transporters (MRP1, BCRP). An MDR1 inhibitor suppressed efflux in murine ovarian cancer cells isolated from ascites *in vivo*, but did not suppress efflux in murine ovarian cancer cells pre-incubated with ascites for 7 days *in vitro*. To explain these differences, we postulate that a component of the ascites microenvironment *in vivo* that is not sustained in ascites culture conditions *in vitro* drives MDR1 efflux function in ovarian cancer cells. Collectively, our results suggest that MRP1 and BCRP are functional efflux proteins in ascites pre-treated ID8 cells.

To validate our findings in human ovarian cancer, we next studied efflux protein expression/function in tumor-derived and ascites-derived human ovarian cancer cell lines. Notably, expression of all three efflux proteins (MDR1, MRP1, BCRP) was elevated in ascites-derived lines compared to the tumor-derived line (Fig 5C). However, only MRP1 and BCRP inhibitors reduced efflux in ascites-derived human ovarian cancer cells (Fig 5D and 5E). We reasoned that the MDR1 inhibitor may not reduce efflux in ascites-derived tumor cells if MDR1 expression is redundant in a setting where MRP1 and BCRP are co-expressed. To address this possibility, we incubated ascites-derived cells simultaneously with three transporter inhibitors (MDR1, MRP1, BCRP). Notably, cells incubated with this combination of inhibitors did not show reduced efflux compared to cells incubated with MRP1 or BCRP inhibitors alone (data not shown). These data suggest that MDR1 protein is not functional in these ascites-derived human ovarian cancer cells; however, we cannot rule out the possibility that these cells express ABC transporters other than MRP1 and BCRP whose function is redundant with that of MDR1. Since BCRP and MRP inhibitors were not synergistic (data not shown), we also hypothesize that MRP1 and BCRP act cooperatively to promote efflux in ascites-derived ovarian cancer cells, a topic for future studies.

The mechanisms by which MDR related transporters are regulated are unclear [24]. Previous induction of chemo-resistance was primarily achieved by culturing cancer cell lines with various chemotherapeutic drugs. Our model is unique in suggesting that an inflammatory component may drive ovarian cancer chemo-resistance, given that ascites supernatant is composed of multiple cytokines released by inflammatory cells. The idea that soluble ascites factors influence ovarian cancer cell function is supported by our previous work, showing that ascites induces ovarian cancer stem cell behaviors in a reversible manner [25]. Correlations have been established between chemo-resistance and levels of cytokines such as interleukin 6 (IL-6) [25] and interleukin 8 (IL-8) [26]. These inflammatory cytokines may be secreted by M2 macrophages, which predict a poor prognosis for advanced epithelial ovarian cancer [27]. Identifying specific cytokines in ascites that drive resistance has the potential to ultimately identify novel therapies that can combat ovarian cancer chemotherapy-resistance.

Our demonstration that ascites drives ABC transporter expression/efflux function in ovarian cancer extends our previous work, showing that ascites enriches for ovarian cancer stem-like cells [25]. Notably, in this previous work, we showed that ascites-enriched murine ovarian cancer cells are characterized by a stemness gene expression signature which includes several ATP transporter genes. The current work extends these findings by showing that these ATP transporter proteins are functional efflux proteins that promote chemotherapy resistance in ascites-associated ovarian cancer cells. Of note, in this previous work we identified cell surface GRP78 as a novel and targetable molecule on these ascites-enriched ovarian cancer stem-like cells. Collectively, our studies underscore the importance of future studies investigating efficacy of targeting cell surface GRP78 in ascites-enriched ovarian cancer stem-like cells to inhibit multidrug resistance, thus increasing chemotherapy efficacy.
